# Regular intake of energy drinks and multivitamin supplements is associated with elevated plasma vitamin B6 levels in post-bariatric patients

**DOI:** 10.1038/s41598-021-97205-7

**Published:** 2021-09-08

**Authors:** Martina Tynes, Matthias Hepprich, Katharina Timper

**Affiliations:** 1 Faculty of Medicine, University of Basel, Klingelbergstrasse 61, 4056 Basel, Switzerland; 2 Clinic of Endocrinology, Diabetes and Metabolism, University Hospital Basel, Petersgraben 4, 4031 Basel, Switzerland; 3 Clinic of Endocrine and Metabolic Disorders, Cantonal Hospital Olten, Basler Strasse 150, 4600 Olten, Switzerland; 4 Department of Biomedicine, University Basel, Hebelstrasse 20, 4031 Basel, Switzerland

**Keywords:** Metabolic diseases, Obesity, Obesity

## Abstract

The aim of the present survey was to analyze plasma vitamin B6 levels in post-bariatric patients and to elucidate the causal factors associated with elevated plasma vitamin B6 levels. This is a retrospective analysis of electronic patient data of all post-bariatric patients evaluated at the endocrine outpatient clinic of the University Hospital Basel in 2017, for which plasma vitamin B6 values were assessed during regular follow-up visits. In total, 205 patients were included in the study, whereof a minority of 43% had vitamin B6 levels in the normal range. 50% of the patients had vitamin B6 levels up to fourfold higher than the upper normal limit and 7% had levels more than fourfold above the upper normal limit. Vitamin B6 deficiency was not observed in any patient. While multivitamin supplementation in general was associated with elevated plasma vitamin B6 levels, the highest vitamin B6 levels were found after biliopancreatic diversion (BPD) and in patients who reported daily energy drink intake. Elevated plasma vitamin B6 levels up to fourfold above the upper normal limit are common in postbariatric patients and are associated with regular multivitamin supplementation, while highly elevated plasma vitamin B6 levels were seen primarily upon regular energy drink intake. Thus, a regular follow-up of vitamin B6 plasma levels and critical evaluation of vitamin B6 supplementation, either as part of the multivitamin preparation or related to regular energy drink intake, is highly warranted and should be an integral part of the routine post-bariatric follow-up.

## Introduction

In 2018, a 30-year old male patient with severe obesity and an initial body-mass-index (BMI) of 75 kg/m^2^ presented to our post-bariatric outpatient clinic with a progressive, immobilizing fatigue as well as par- and dysesthesia of the fingers IV and V of the left hand with a reduced amplitude of the sensory nerve action potential of the left ulnar nerve as revealed upon electroneurography. This patient had previously received subsequent bariatric procedures such as sleeve gastrectomy (LSG), followed by omega-loop bypass and biliopancreatic diversion (BPD) over a time span of 4 years, leading to substantial weight loss with a current BMI of 49.3 kg/m^2^. Laboratory analysis revealed massively elevated plasma vitamin B6 levels (up to 1600 nmol/l, normal reference range 35–110 nmol/l), which had been present for several years in retrospect. Medical history of the patient unveiled an intake of 2–3 energy drinks per day (10–15 mg vitamin B6 per day) in addition to his regular multivitamin supplementation (2 mg vitamin B6 per day). After complete withdrawal of energy drinks and replacement of the multivitamin by a supplement with reduced vitamin B6 content, vitamin B6 levels decreased to 400 nmol/l and clinical symptoms of fatigue and neuropathy resolved completely.

Vitamin B6, also known as pyridoxine, is a water-soluble vitamin present in various food products as well as in dietary supplements^1^. Vitamin B6 comprises a group of the interrelated vitamers pyridoxine, pyridoxal (PL) and pyridoxamine and their respective monophosphorylated derivatives, which are converted to the biologically active form pyridoxal 5′-phosphate (PLP)^2^.

Fasting plasma PLP concentrations are most accurately reflecting vitamin B6 body stores^3^ and systemic vitamin B6 levels are commonly assessed by direct detection of PLP concentration in plasma as it reflects PLP concentrations of the liver, and thus, vitamin B6 intake over time^2^.

Swiss recommendations regarding the daily intake of vitamin B6 in adults are 1.4 mg/day for women and 1.6 mg/day for men with slightly different recommendations for infants, children, adolescents, pregnant and breast-feeding women^4^. Daily vitamin B6 intake should not exceed 25 mg as an intake of 50 mg/day is associated with neurological side effects^5,6^. Thereby, pyridoxine, the main B6 vitamer in dietary supplements^7^, is supposed to be the main driver of vitamin B6 toxicity^5^. Elevated plasma vitamin B6 levels are associated with sensory and peripheral neuropathy, leading to progressive sensory ataxia, unstable gait, numbness of the hands and absent tendon reflexes^8^. Furthermore, photosensitivity^9^ and mild motor neuropathy^10^ have been reported.

Of note, both vitamin B6 intoxication and vitamin B6 deficiency can lead to neuropathy, as in both cases PLP-dependent enzymes are inhibited by pyridoxine^5^. Vitamin B6 deficiency is defined by a serum PLP level < 20 nmol/l^11^ and associated with an increased risk for cardiovascular disease^12^, depression, impaired immune response as well as sideroblastic anemia, seizures and convulsions^2,13,14^. Following bariatric surgery, general daily multivitamin supplementation, containing vitamin B6, is recommended by various society guidelines^15^.

As we diagnosed a severe symptomatic vitamin B6 hypervitaminosis in one of our post-bariatric patients, we aimed at retrospectively analyzing plasma vitamin B6 levels in all post-bariatric patients assessed at our obesity outpatient clinic during the year 2017 and to elucidate the underlying factors associated with elevated plasma vitamin B6 levels.

## Results

### Baseline characteristics of the study population

**Figure 1:**
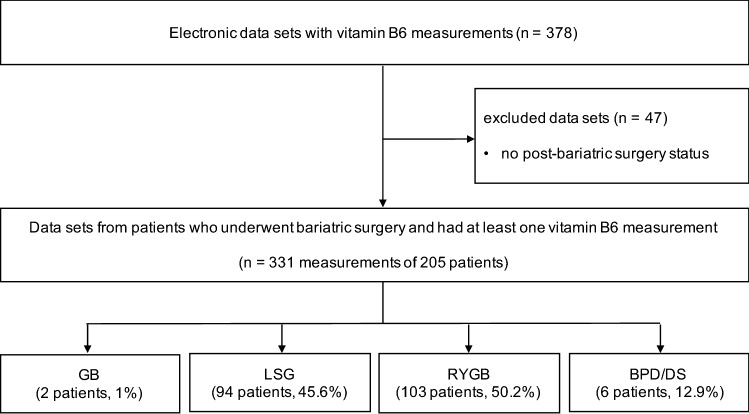
Study flow chart. *RYGB* Roux-en-Y gastric bypass, *LSG* laparoscopic sleeve gastrectomy, *GB* gastric banding, *BPD* biliopancreatic diversion, *DS* duodenal switch, *n* number of measurements.

Our electronic data search identified 378 vitamin B6 data sets from 236 patients visiting the bariatric outpatient clinic of the University Hospital Basel, Basel, Switzerland, in 2017. Thereof, 47 measurements from 31 patients were excluded due to the lack of post-bariatric status. Thus, the final patient population comprised 205 patients (103 Roux-en-Y gastric bypass [RYGB], 94 laparoscopic sleeve gastrectomy [LSG], 6 biliopancreatic diversion [BPD] or duodenal switch [DS] and 2 gastric banding [GB]) with a total of 331 vitamin B6 measurements in 2017 as shown in Fig. 1. Due to the regular routine schedule of patients after bariatric surgery, some patients had more than one check-up of their nutritional status and thus more than one vitamin B6 measurement during the investigated time period.

Baseline characteristics of the investigated study population are depicted in Table 1. Patients were mostly female (n = 148, 72.2%), had a median age of 46 years and a median postoperative interval of 23 months since the last bariatric surgery procedure. The median weight was 87 kg and median body mass index (BMI) was 32 kg/m^2^. Parameters for blood pressure and heart rate were in the normal range.

**Table 1:** Baseline characteristics.

Characteristic	Median (IQR; range)	n (%)
Total patients		205 (100%)
Age (years)	46 (36–57; 20–73)	205 (100%)
**Sex**		
Male		57 (27.8%)
Female		148 (72.2%)
Height (cm)	167 (160–173; 152–196)	164 (80%)
Weight (kg)	87 (76.5–104; 50–206)	164 (80%)
BMI (kg/m^2^)	32 (28.3–36.3; 20.2–60.2)	164 (80%)
Systolic blood pressure (mmHg)	121 (110–134; 75–175)	157 (76.6%)
Diastolic blood pressure (mmHg)	76 (69.5–87; 40–115)	157 (76.6%)
Heart rate (bpm)	72 (65–83; 42–130)	157 (76.6%)
**Type of bariatric surgery**		
Gastric banding		2 (1%)
Sleeve Gastrectomy		94 (45.6%)
Roux-Y-gastric bypass		103 (50.2%)
Biliopancreatic diversion		6 (2.9%)
Postoperative interval (months)	23 (4–39; 1–288)	205 (100%)
**Laboratory analyses**		
Vitamin B1 (nmol/l) [reference 66–200 nmol/l]	150 (129–181; 24–277)	331 (100%)
Vitamin B6 (nmol/l) [reference 35–110 nmol/l]	132 (97–208; 40–798)	331 (100%)
Vitamin B12 (pmol/l) [reference > 200 pmol/l]	303 (224–420; 99–1476)	320 (96.7%)
Folic acid (nmol/l) [reference 8.8–60.8 nmol/l]	20 (11–32; 4.5–46)	317 (95.8%)
25-hydroxyvitamin D (nmol/l) [reference 50–70 nmol/l]	61 (43–81; 10–145)	321 (97%)
Ferritin (µg/l) [reference 10–200 μg/l]	67 (28–128; 6–1073)	318 (96.1%)

Baseline characteristics of all patients after bariatric surgery with regular vitamin B6 measurements in 2017.

*bpm* beats per minute.

### No case of vitamin B6 deficiency and elevated plasma vitamin B6 levels in 57% of all investigated post-bariatric patients

First, we analyzed the plasma vitamin B6 levels in our study population. Importantly, none of the patients presented with vitamin B6 deficiency, independent from regular intake of multivitamin supplementation. Moreover, vitamin B6 levels in the normal range were found in only 43.4% of the study population, while 56.6% of all patients presented with vitamin B6 levels above the upper normal limit, whereof plasma vitamin B6 levels were increased up to fourfold in 49.8% of the patients and more than fourfold above the upper normal limit in 6.8% of the patients (data not shown).

### Time after bariatric surgery procedure does not impact plasma vitamin B6 levels

**Figure 2:**
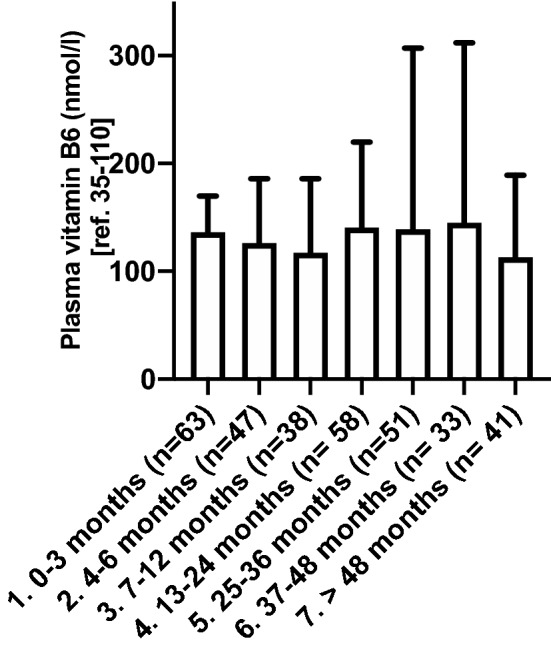
Plasma vitamin B6 levels according to postoperative time interval from last bariatric surgery procedure. Vitamin B6 plasma levels at different time points after the last bariatric surgery procedure. Data are presented as median plasma levels and interquartile range. There was no significant difference between the groups. *n* number of vitamin B6 measurements in 205 patients in 2017.

Next, we investigated, whether the time span after the last bariatric surgery procedure impacted plasma vitamin B6 levels in our study population. Median vitamin B6 plasma levels in patients after different time intervals post-surgery did not differ from each other (Fig. 2). Thus, time span after bariatric surgery procedure did not influence plasma vitamin B6 levels in our patient population.

### Malabsorptive bariatric procedures are associated with higher plasma vitamin B6 levels

**Figure 3:**
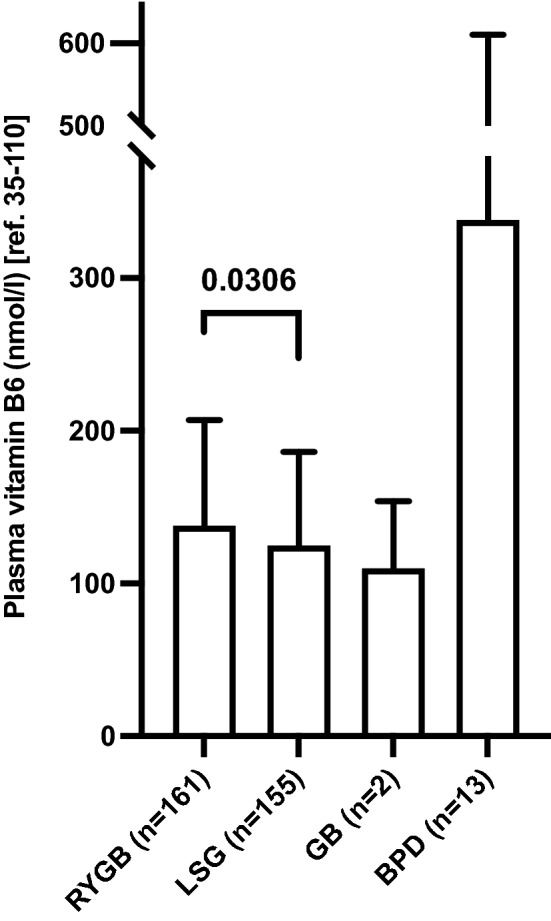
Plasma vitamin B6 levels in patients after different bariatric surgery procedures. Data are presented as median plasma vitamin B6 levels and interquartile range (IQR) for each bariatric surgery procedure. Mean values were 176 nmol/l (IQR 104–205), 161 nmol/l (IQR 87–186), 110 nmol/l (IQR 88–132), and 347 nmol/l (IQR 102–577) in patients with Roux-en-Y gastric bypass (RYGB), laparoscopic sleeve gastrectomy (LSG), gastric banding (GB), and biliopancreatic diversion (BPD). Kruskal–Wallis-Test for intergroup comparison was significant (p-value 0.033). Level of significance between RYGB and LSG was calculated with Mann–Whitney U-test and is indicated in the graph with horizontal bar. Level of significance was not calculated for differences between gastric bypass (GB) or biliopancreatic diversion (BPD) due to low sample size. *n* number of plasma vitamin B6 measurements in 205 patients in 2017.

Following, we asked whether plasma vitamin B6 level were associated with the type of bariatric procedure. Median plasma vitamin B6 levels were 138 nmol/l in patients after RYGB, 125 nmol/l after LSG, 110 nmol/l after GB and 338 nmol/l after BPD, respectively (Fig. 3). Thus, plasma vitamin B6 levels upon malabsorptive procedures were significantly higher than upon more restrictive procedures such as LSG, whereby the highest levels were found upon BPD.

### Regular multivitamin supplement intake is associated with elevated plasma vitamin B6 levels

**Figure 4:**
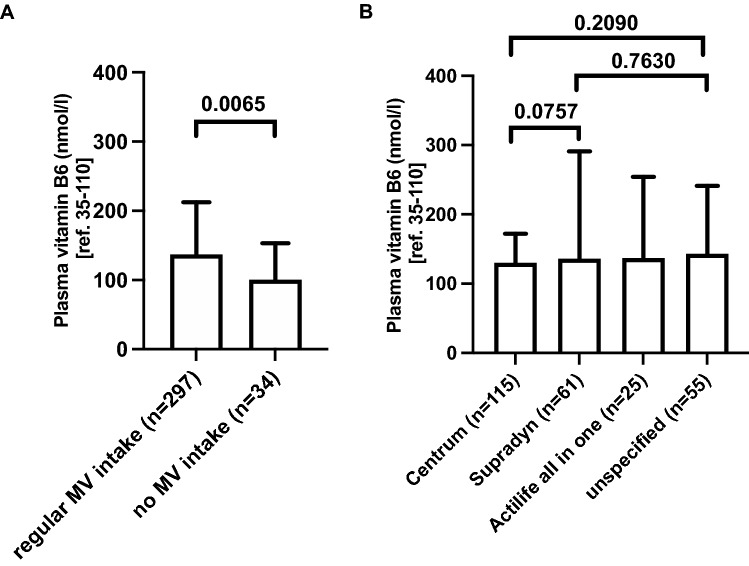
Plasma vitamin B6 levels and multivitamin supplementation intake in post-bariatric patients. (**A**) Presented are median plasma vitamin B6 levels and interquartile ranges (IQR) in patients with regular multivitamin (MV) intake (137 nmol/l, IQR 99–213) compared to patients who reported no regular multivitamin supplementation (101 nmol/l, IQR 73–153). (**B**) Plasma vitamin B6 levels in patients with recorded intake of the most common multivitamin compounds compared to those with recorded intake of multivitamin supplements without specification of the name of the compound (“unspecified”). Data are presented as median and interquartile ranges. Mean values were 145 nmol/l (IQR 102–172), 216 nmol/l (IQR 99–285), 210 nmol/l (IQR 101–221), and 202 nmol/l (IQR 96–241) in patients taking “Centrum von A bis Zink” (vitamin B6 content: 2 mg per serving), “Supradyn energy” (vitamin B6 content: 4 mg per serving), “Actilife all in one Depot” (vitamin B6 content: 1.4 mg per serving), and unspecified multivitamin compounds, respectively. Kruskal–Wallis-Test for intergroup comparison was not significant (p-value 0.28). Level of significance between two groups was calculated with Mann–Whitney U-test and is indicated in the graph with horizontal bars. *n* number of plasma vitamin B6 measurements in 205 patients in 2017.

Next, we intended to unravel the impact of regular multivitamin intake on plasma vitamin B6 levels in our patients. Regular multivitamin intake was associated with significantly higher median plasma vitamin B6 levels compared to no intake (137 nmol/l, IQR 99–213 vs. 101 nmol/l, IQR 73–153, Fig. 4A). In patients without regular multivitamin intake, plasma vitamin B6 levels ranged 42–457 nmol/l and thus were all above the lower reference limit of 35 nmol/l.

Following, we investigated if and how plasma vitamin B6 levels were impacted by different multivitamin supplements. Surprisingly, although the amount of vitamin B6 differs between the multivitamin supplements, ranging from 1.4 to 4 mg per serving for the most common compounds, plasma vitamin B6 levels were not significantly different between the groups (Fig. 4B).

Vitamin B6 plays an important role in methionine metabolism together with vitamin B1, B2, B12, homocysteine and folic acid^16^. Vitamin B1, B12 and folic acid are recommended to be routinely supplemented in postbariatric patients^15^ and are part of common multivitamin supplements. As we found overall higher plasma vitamin B6 levels in patients upon regular multivitamin intake, we aimed to analyze if plasma levels of vitamin B6 correlated with plasma levels of vitamin B1, B12 and folic acid. We observed significant mild correlations of vitamin B6 with vitamin B1, B12 and folic acid in our patients (Fig. S1), indicating that micronutrient status may be partly reflected by either of the mentioned substrates. Although we found elevated vitamin B1 levels (maximum up to twofold above the upper normal limit in 48 patients (14.5%)) the prevalence was much lower compared to patients presenting with elevated vitamin B6 levels. Regarding folic acid, the upper assay limit of 45 nmol/l hindered to assess whether plasma levels were above the upper reference level of 60.8 nmol/l which was the case in only 35 patients (10.5%). There is no upper limit for plasma vitamin B12 levels. Vitamin B12 levels were below 200 pmol/l in 53 patients (16%) of which 15 patients (4.5%) had levels below 150 pmol/l, indicating vitamin B12 deficiency. However, vitamin B12 is commonly applied parenterally in bariatric patients and thus vitamin B12 plasma level do not accurately reflect oral substitution.

### Regular energy drink intake is associated with increased plasma vitamin B6 levels

**Figure 5:**
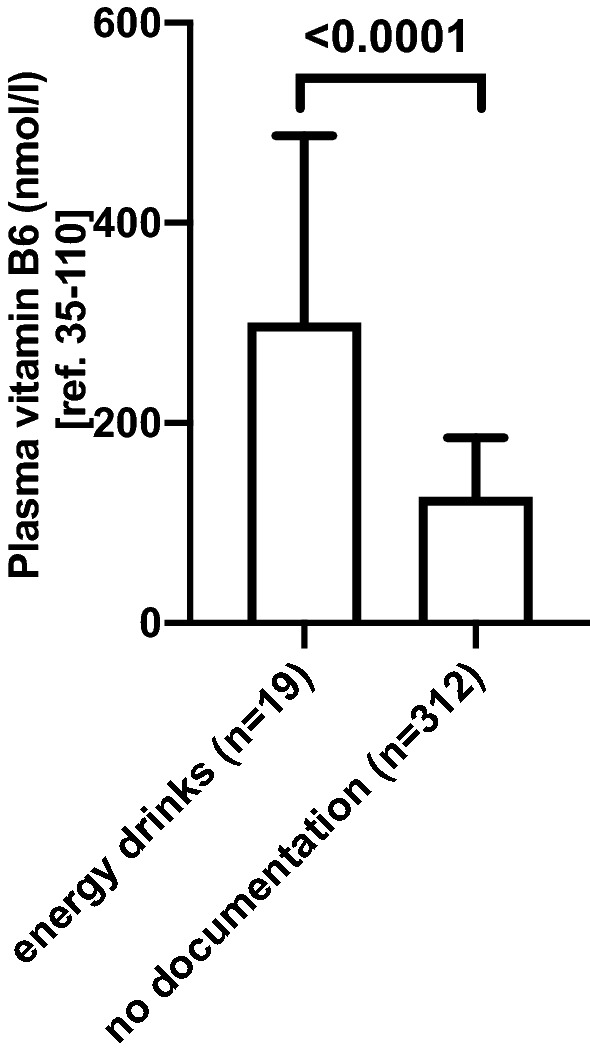
Plasma vitamin B6 levels in postbariatric patients with documented energy drink consumption. Vitamin B6 plasma level measurements in patients (n = 12) with reported energy drink intake compared to patients without documented energy drink intake (n = 193). Data are presented as median with interquartile range. Level of significance between the two groups was calculated with Mann–Whitney U-test. *n* number of plasma vitamin B6 measurements in 205 patients in 2017.

Energy drinks contain substantial amounts of vitamin B6, ranging from 2 to 40 mg per serving depending on the individual product^17^. The finding of a severe symptomatic vitamin B6 hypervitaminosis in one of our post-bariatric patients upon excessive energy drink intake prompted us to investigate if and how energy drink intake impacted plasma vitamin B6 levels in our post-bariatric patient cohort. Regular energy drink intake was recorded in 12 patients. Of note, pyridoxine levels of common energy drinks in Switzerland range from 1.99 mg (“Prix Garantie Energydrink”) to 5 mg (“Redbull”) per serving. Interestingly, plasma vitamin B6 levels were increased to a median level of 300 nmol/l (IQR 190–487) in patients reporting regular energy drink intake (12 patients) compared to 126 nmol/l (IQR 95–185) in patients without documented energy drink consumption (193 patients) (Fig. 5).

### Patients with neurological symptoms had a wide range of plasma vitamin B6 levels

Finally, we assessed plasma vitamin B6 levels in post-bariatric patients presenting with neurological symptoms such as sensory polyneuropathy, fatigue, sulcus ulnaris syndrome and numbness. In total, neurological symptoms were documented in 11 patients, whereof 3 patients reported hypoesthesia, 3 reported paresthesia, 3 reported pain, 3 reported fatigue and 1 patient reported cubital tunnel syndrome of unknown origin. These patients had a median plasma vitamin B6 level of 150 nmol/l, ranging from 65–661 nmol/l. Among those, vitamin B6 levels were in the normal range (35–10 nmol/l) in 3 patients, up to fourfold increased (110–440 nmol/l) in 7 patients and increased more than fourfold above the upper normal limit (> 440 nmol/l) in 1 patient, our index case.

## Discussion

Our study revealed elevated plasma vitamin B6 levels in 2/3 of our post-bariatric patients, while only 1/3 presented with levels within the normal range. Plasma vitamin B6 levels were found to be increased more than fourfold above the upper normal limit in 6.8% of the patients. Of note, no case of vitamin B6 deficiency was found in this cohort. We found elevated plasma vitamin B6 levels to be associated with regular multivitamin and energy drink intake.

Our findings are in line with other reports describing elevated plasma vitamin B6 levels in postbariatric patients upon regular multivitamin intake^16,18–24^, challenging the assumption that the postbariatric status per se is associated with a risk for vitamin B6 deficiency. This is supported by the observation that postbariatric vitamin B6 deficiency was mainly reported in patient collectives where a deficiency was already present before surgery^13,25,26^.

In particular, patients without regular multivitamin supplementation presented with median plasma vitamin B6 levels in the normal range, whereas regular multivitamin supplement intake was associated with higher median plasma vitamin B6 levels. Interestingly, the exact dose of daily vitamin B6 intake, ranging from 1.4 to 6 mg vitamin B6 depending on the type of supplement, had no impact on plasma vitamin B6 levels. A recent report by Heusschen et al. demonstrated increased vitamin B6 levels after LSG in almost 1/3 of the study population^27^. In line with our findings, Heusschen et al. found no correlation of the type of multivitamin supplementation with the prevalence of B6 hypervitaminosis^27^. Moreover, Cupa et al. reported a serious case of symptomatic vitamin B6 intoxication in a patient after BPD associated with excessive daily vitamin B6 supplementation of 300 mg for 6 months with full resolution of all symptoms after vitamin B6 supplementation was omitted^28^.

All patients included in the study received the same postbariaric nutritional counseling including a protein intake of 1.2 g/kg body weight/day, daily multivitamin supplementation and an oral calcium intake of > 1000 mg/day upon sleeve gastrectomy and > 1500 mg/day upon gastric bypass and biliopancreatic diversion. Detailed analyses of the nutritionists’ records of our patients presenting with more than fourfold elevated vitamin B6 plasma levels revealed 8/14 patients with regular meal rhythm and 3 main meals and 1–3 snacks while 2/14 patients reported a regular meal rhythm with 1 main meal and 1–3 snacks and 1/14 patients mainly taking formula diet. From 3/14 patients no information was available. Regarding nutritional calcium intake, 6/14 patients reported sufficient and 6/14 patients reported insufficient intake while no information was available from 2/14 patients. Nutritional protein intake was recorded sufficient in 3/14 patients and insufficient in 8/14 patients while no information was available from 3/14 patients. Regarding the composition of the food, regular/daily intake of the following food items were quite heterogeneous and were reported as follows: Milk 6/14; Yoghurt 5/14; Meat 6/14; Vegetables 2/14; Cheese 6/14; Eggs 6/14; Fruits 1/14; Nuts 1/14; Pasta 1/14; Coffee 3/14.

Overall, nutritional information is not complete and difficult to assess, especially in retrospect. However, we did not find records of any special food additives in patients with very high plasma vitamin B6 levels other than regular multivitamin supplements and regular energy dink intake. Of note and most importantly, vitamin B6 is found in natural food components at low levels (0.01–1.0 mg/100 g)^29^. We cannot exclude that an increase in meat intake, as a natural vitamin B6 source^29^, contributed to increased vitamin B6 intake. However, only 6/14 patients reported regular meat intake and sufficient intake of protein was only recorded in 3/14 patients. Detailed analyses of the nutritionists’ records of our patients presenting with more than fourfold elevated vitamin B6 plasma levels and comparing data with the Swiss Food Composition Database^29^ did not identify natural food products or fortified substances other than multivitamins (daily intake by 13/14 patients) and energy drinks (daily intake by 5/14 patients) that would explain excessive vitamin B6 levels observed in this patient group.

While supplementation of various macro- and micronutrients is important to prevent deficiencies after bariatric surgery^30^ and recommended by various society guidelines^15^, a substantial body of evidence points towards an over-supplementation of post-bariatric patients by daily vitamin B6 intake^16,18–24,27,28^. Thus, prospective, multicenter studies are needed to unravel, if multivitamin formulations recommended to post-bariatric patients should be adapted regarding their vitamin B6 content to avoid over-supplementation and possible associated side effects in the future.

In contrast to multivitamin intake, time span after surgical procedure did not impact plasma vitamin B6 levels in this cohort which is in line with a recent study reporting elevated plasma vitamin B6 levels after 5 years post-surgery compared to pre-operative levels^19^.

We found, that malabsorptive bariatric procedures like RYGB and BPD were associated with higher plasma vitamin B6 levels than restrictive procedures, such as GB and LSG. Upon RYGB and BPD and thus bypassing a significant part of the stomach, the duodenum and the upper jejunum, the absorption and metabolism of certain vitamins and micronutrients is usually impaired^31^. Vitamin B6 is derived not only from dietary but also from bacterial sources such as the microbiome of the large intestine^32^. Magnúsdóttir et al. reported that several microbiota are able to synthesize PLP^33^ and that up to 86% of the recommended daily vitamin B6 intake can be produced by gut microflora. Thus, alterations of the gut microbiota composition after bariatric surgery^34–37^, might contribute to the increase in availabe vitamin B6 derived from gut microflora. In line with this hypothesis, Guo et al. reported an increase in Bacteroidetes and Proteobacteria phyla after bariatric surgery^34^, which are both able to synthesize PLP^33^. Thus, changes in the gut microbiome composition after bariatric surgery might raise PLP content in the large intestine in post bariatric patients.

Our most important finding is, that the regular intake of energy drinks is associated with increased plasma vitamin B6 levels in post-bariatric patients. Although the vitamin B6 amount of common energy drinks differs quite substantially (1.99–5 mg per serving), all clearly exceed the recommended daily amount of vitamin B6 intake in Switzerland^4^. The identification of energy drinks as a high vitamin B6 source is of high clinical relevance and should raise the awareness of health care providers to carefully evaluate and advise against energy dink intake in postbariatric patients to prevent vitamin B6 intoxication and possible associated neurological side effects.

In our study, 11 patients reported neurological symptoms of unknown origin whereby all of these patients had plasma vitamin B6 levels above the upper normal limit. The reported symptoms were mainly fatigue and peripheral neuropathy, both known to be associated with vitamin B6 intoxication^8,38^. However, except for our index patient case, where a complete withdrawal of energy drinks and replacement of the multivitamin by a supplement with reduced vitamin B6 content resulted in a fourfold decrease of the plasma vitamin B6 levels and a complete resolution of all neurological symptoms, a direct correlation between elevated vitamin B6 levels and neurological symptoms in the other patients remains elusive. Already in 1983 Schaumburg et al. reported seven cases of patients with polyneuropathy and ataxia as well as severe sensory-nervous-system dysfunction upon daily pyridoxine intake of 2–6 g during 2–40 months^8^. Another study depicted a case of severe sensory and mild motor neuropathy due to intake of 10 g vitamin B6 per day over 5 years^10^. Thus, vitamin B6 intoxication with neurologic symptoms has been reported in non-bariatric patients upon excessive daily vitamin B6 intake over a longer time span. In keeping with our findings, previous studies demonstrated symptomatic vitamin B6 intoxication in post-bariatric patients already at much lower daily vitamin B6 doses^28,39^. In line, Janssen et al. reported one case of peripheral neuropathy possibly related to vitamin B6 intoxication in a post-bariatric patient and full relieve of the neurologic symptoms upon discontinuation of the vitamin B6 supplementation^20^. In summary, neurological symptoms such as neuropathy might be associated with vitamin B6 intoxication in post-bariatric patients and should prompt healthcare providers to carefully evaluate plasma vitamin B6 levels and intake in these patients.

This study has several limitations. The retrospective analysis of patient records of a single-center in Switzerland with a relatively small number of patients, especially with regard to the subgroups, may not allow full translation of our findings to bariatric centers in other countries given the large variety of possibly impacting socioeconomic, geographic/climatic and nutritional factors. In addition, in some patients plasma vitamin B6 level were assessed more than once during the evaluation period due to the regular schedule of post-surgical follow-up visits. Therefore, changes over time were not detected accurately. Furthermore, no comparison with different ethnicities was made.

Plasma vitamin B6 levels are affected by impaired kidney function, low plasma albumin, glucose levels, inflammation and elevated alkaline phosphatase (all associated with reduced plasma vitamin B6 levels)^7^, factors which we did not control for in our survey. Thus, actual plasma vitamin B6 levels might be even higher than reported in the present study.

However, the present study provides a thorough analysis of vitamin B6 levels and uncoveres nutritional factors leading to the high frequency of elevated plasma vitamin B6 levels in post-bariatric patients in a Swiss tertiary care hospital. Our data questions the current dogma, which is that the post-bariatric state is per se associated with vitamin B6 deficiency. Thus, the general recommendations for vitamin B6 supplementation in all post-bariatric patients should be revised towards individualized, tailored approaches according to actual plasma vitamin B6 levels in these patients. Accordingly, the composition of multivitamin supplements recommended to post-bariatric patients should be adjusted to lower or no vitamin B6/pyridoxine content and thus should prompt the development of specific multivitamin formulations for post-bariatric patients.

Our study is the first to uncover regular energy drink intake as an important contributor to vitamin B6 intoxication in patients after bariatric surgery. This finding is of high clinical relevance for all health care providers involved in the follow up of bariatric patients and should prompt careful evaluation and advise against energy dink intake in postbariatric patients to prevent vitamin B6 intoxication and possible associated neurological side effects.

## Conclusion

In summary, elevated vitamin B6 plasma levels are common in patients after bariatric surgery and are associated with regular multivitamin as well as energy drink intake. As vitamin B6 oversupply may lead to neurological side-effects, vitamin B6 levels shall be carefully monitored in postbariatric patients. Patients after bariatric surgery should receive a tailored supplementation of vitamin B6 according to their needs and should be advised to abstain from energy drink intake to avoid vitamin B6 intoxication and possible side effects.

## Methods

### Study design, setting and participants

This is a retrospective cohort study and quality assurance analysis of all post-bariatric patients visiting the bariatric outpatient clinic of the Endocrinology Department at the University Hospital Basel in 2017. Data was derived upon an electronic search from our electronic patient data base (ISMED) on November 11th 2018 including all post-bariatric patients visiting our endocrine outpatient clinic including assessment of plasma vitamin B6 levels between January 1st 2017 and December 31st 2017. All hits were screened for bariatric procedure, time since surgery, multivitamin supplementation, consumption of energy drinks and neurological symptoms. Inclusion criteria were one or more bariatric procedures before or in 2017 and at least one plasma vitamin B6 measurement in 2017 assessed during a post-surgery follow-up appointment.

### Vitamin B6 measurement

Plasma vitamin B6 levels were assessed via measurement of PLP from venous EDTA blood samplings by high-pressure liquid chromatography (HPLC) at the laboratory of Viollier, Basel, Switzerland. PLP levels between 35 and 110 nmol/l were defined as normal. The study was approved by the local Ethics committee “Nordwest- und Zentralschweiz”, Basel, Switzerland (EKNZ 2019-02216). All methods were performed in accordance with the relevant guidelines and regulations. Data was collected retrospectively as a quality control survey in our department. As the data was classified for further use of previously collected biological material and health-related personal data by the local Ethics committee, informed consent was not required according to Art. 34. HFG, Art. 37–40 HFV.

### Assignment of patients to subgroups

First, vitamin B6 levels were assessed according to the type of bariatric surgery and second, according to the post-operative time span. Third, patients were assigned to groups according to the type of their daily intake of multivitamin preparation. Forth, the patients were assigned to groups according to recorded energy drink intake. Fifth, patients were assigned to groups according to documented neurological symptoms.

### Statistical analysis

The primary outcome was to assess plasma vitamin B6 levels in a cohort of post bariatric patients. Statistical analyses are explanatory, and no sample size calculation was done. Descriptive statistics was used to assess baseline characteristics, the number of vitamin B6 values in the reference range, up to fourfold, and more than fourfold above upper-reference limit for all post-bariatric patients as well as for each bariatric procedure, the postoperative time span and intake of multivitamins and energy drinks. Missing data were not imputed.

In all figures, data are expressed as median and interquartile range. Normality was assessed by comparing median, mean, skewness and kurtosis as well as using Shapiro–Wilk-Test. If no normality was found, non-parametric tests were used. For comparisons between more than 2 groups Kruskal–Wallis test was used. When only 2 groups were compared Mann–Whitney U-test was used for calculating level of significance. Spearman’s rank correlation test was used for comparing non-parametric variables. All statistical analyses were 2-sided and a p-value of < 0.05 was considered statistically significant.

All statistical analyses were done using Graph Prism Version 8.2.1 for macOS.

## Supplementary Information

Supplementary Information.

## Data Availability

The datasets analyzed during the current study are available from the corresponding author on reasonable request.

## Abbreviations

BMI: Body mass index
BPD: Biliopancreatic diversion
GB: Gastric banding
DS: Duodenal switch
IQR: Interquartile range
LSG: Laparoscopic sleeve gastrectomy
PL: Pyridoxal
PLP: Pyridoxal 5′-phosphate
RYGB: Roux-en-Y gastric bypass
