# Supplementary material for: Regular intake of energy drinks and multivitamin supplements is associated with elevated plasma vitamin B6 levels in post-bariatric patients
Source: Sci Rep. 2021 Sep 8;11:17830. doi: 10.1038/s41598-021-97205-7 (PMC8426371; doi:10.1038/s41598-021-97205-7)
Supplement: Supplementary file 1 — Supplementary Information. [file 41598_2021_97205_MOESM1_ESM.pdf]

Regular intake of energy drinks and multivitamin supplements is associated with elevated plasma vitamin B6 levels in post-bariatric patients

Supplementary Figure S1

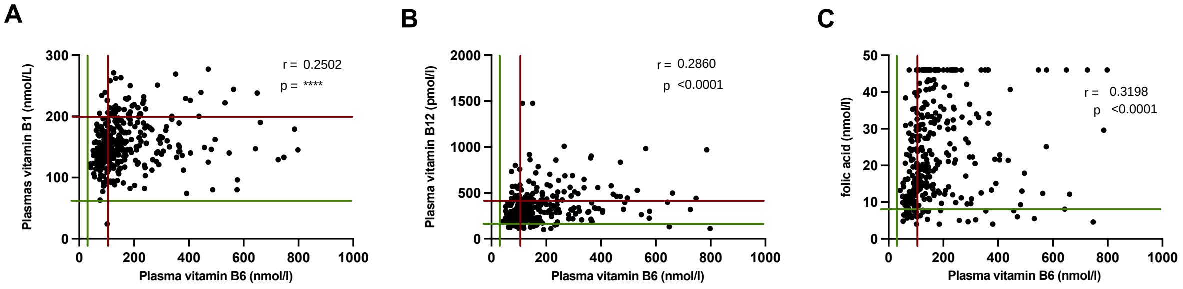

Supplementary Figure 1. Plasma vitamin B6 levels in postbariatric patients and respective levels of [A] vitamin B1 (n=313 pairs), [B] vitamin B12 (n=320 pairs), and [C] folic acid (n=317 pairs) in 205 patients in 2017. Correlation was calculated using Spearman's R. p = level of significance. Green and red line indicate lower and upper reference limit. Of note, upper assay limit of folic acid assay is 45 nmol/l while the upper reference limit is 60.8 nmol/l.
